# Ethics of Wearable-Based Out-of-Hospital Cardiac Arrest Detection

**DOI:** 10.1161/CIRCEP.124.012913

**Published:** 2024-08-22

**Authors:** Marijn Eversdijk, Mirela Habibović, Dick L. Willems, Willem J. Kop, M. Corrette Ploem, Lukas R.C. Dekker, Hanno L. Tan, Rik Vullings, Marieke A.R. Bak

**Affiliations:** 1 Department of Medical and Clinical Psychology, Center of Research on Psychological Disorders and Somatic Diseases, Tilburg University, the Netherlands (M.E., M.H., W.J.K.).; 2 Department of Ethics, Law and Humanities (M.E., D.L.W., M.C.P., M.A.R.B.), Amsterdam UMC, University of Amsterdam, the Netherlands.; 3 Department of Clinical and Experimental Cardiology (H.L.T.), Amsterdam UMC, University of Amsterdam, the Netherlands.; 4 Department of Electrical Engineering, Eindhoven University of Technology, the Netherlands (L.R.C.D., R.V.).; 5 Department of Cardiology, Catharina Hospital, Eindhoven, the Netherlands (L.R.C.D.).; 6 Netherlands Heart Institute, Utrecht (H.L.T.).

**Keywords:** beneficence, digital health, ethics, health equity, informed consent

## Abstract

Out-of-hospital cardiac arrest is a major health problem, and immediate treatment is essential for improving the chances of survival. The development of technological solutions to detect out-of-hospital cardiac arrest and alert emergency responders is gaining momentum; multiple research consortia are currently developing wearable technology for this purpose. For the responsible design and implementation of this technology, it is necessary to attend to the ethical implications. This review identifies relevant ethical aspects of wearable-based out-of-hospital cardiac arrest detection according to four key principles of medical ethics. First, aspects related to beneficence concern the effectiveness of the technology. Second, nonmaleficence requires preventing psychological distress associated with wearing the device and raises questions about the desirability of screening. Third, grounded in autonomy are empowerment, the potential reidentification from continuously collected data, issues of data access, bystander privacy, and informed consent. Finally, justice concerns include the risks of algorithmic bias and unequal technology access. Based on this overview and relevant legislation, we formulate design recommendations. We suggest that key elements are device accuracy and reliability, dynamic consent, purpose limitation, and personalization. Further empirical research is needed into the perspectives of stakeholders, including people at risk of out-of-hospital cardiac arrest and their next-of-kin, to achieve a successful and ethically balanced integration of this technology in society.

Out-of-hospital cardiac arrest (OHCA) is a major health problem, with a yearly incidence of ≈40 to 100 individuals per 100 000 in the global population and a chance of survival ranging between 4.6% and 16.4%.^1–3^ Current research efforts focus on technological solutions to increase the chances of survival.^4^ A new technique under development is the use of wearable technology to detect and alert emergency responders in the case of OHCA. Wearable technology is defined as a set of technological devices (e.g., smartwatches, rings, patches, and wristbands) that can be worn for continuous monitoring of an individual’s physiological biomarkers.^5,6^ By designing wearable technology to act as an automated first witness of OHCA, this technology would enable the start of the chain of survival in unwitnessed situations (≈40% of all OHCA cases) while also saving valuable time in witnessed cases of OHCA.

Several research consortia are currently developing a system in which a permanently worn wearable device autonomously detects OHCA and subsequently sends an alert to emergency responders, such as the DETECT, HEART-SAFE, and Beating Cardiac Arrest (BECA) projects.^7–10^ Whether the OHCA detection technology should be a stand-alone wearable device or an algorithm integrated into existing smartwatch technology is still up for debate, but the first empirical data from the DETECT-1 study on a wearable device already showed promising results in terms of high sensitivity and a relatively low number of false positives in a clinical setting.^10^ However, there has been little attention to the ethical, legal, and social issues associated with this wearable-based OHCA detection, such as privacy concerns related to monitoring and location tracking. This is an important gap because wearable-based OHCA detection will only successfully increase the likelihood of survival after OHCA if the technology is broadly accepted and integrated into society. An example of the development of a wearable-based OHCA detection system is the BECA project, in which several of the authors participate and ethical issues will be systematically addressed^9^ (Figure 1). Identifying the ethical, legal, and social issues of wearable-based OHCA detection in BECAand similar ongoing projects is needed to maximize acceptability and decrease potential distress among users, including wearers of the device, bystanders, and health workers.

**Figure 1.**
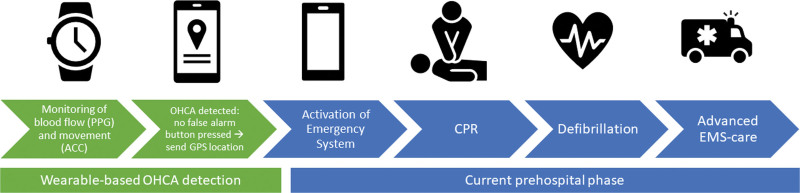
**Schematic illustration of the addition of a wearable (as developed in the Beating Cardiac Arrest project) to the prehospital phase of the out-of-hospital cardiac arrest (OHCA) chain of survival.** The wearable technology continuously tracks local variations in blood volume and movement through the use of photoplethysmography (PPG) and accelerometer (ACC) sensors. When both sensors indicate the potential presence of a cardiac arrest, the technology will set off a local tactile/audible alarm to allow the wearer to cancel in the case of a false alarm. When there is no response to the local alarm, the wearer is assumed to be unconscious because of a cardiac arrest. An alert with a GPS-based position will be sent to the emergency dispatch center, which will send the location to the emergency medical services (EMS). Emergency responders are both professionals (e.g., ambulance personnel) and citizen responders from the existing Dutch citizen responder system (HartslagNu). They will receive the geographic location of the potential OHCA victim and will be asked to perform basic life support and defibrillation using an automated external defibrillator. Figure adapted from Thannhauser et al^11^ with permission.

An essential step in moving this wearable technology forward is exploring the ethics part of ethical, legal, and social issues, which forms the basis for further legal analyses and empirical studies investigating psychosocial impacts. Due to the passive and continuous health data collection from wearable technology, there is a lot of emphasis on data ethics in the literature. Other review articles have previously identified general ethical issues with passive health data collection (e.g., through smartwatches), including concerns around privacy, informed consent, data security, equal access, and ownership.^12^ While these aspects apply to health-targeting wearables in general, a more specified approach is warranted to move beyond general data protection concerns and evaluate the particular ethics of wearable-based OHCA detection (e.g., the immediate action required to act on an alarm). This inventory of specific challenges is necessary to successfully embed ethics in the design of new technologies.^13^ In this review, we assess the ethical challenges with the use of wearables in health care and how these apply to wearable-based OHCA detection. We categorize themes according to four key principles in medical ethics and provide specific design recommendations while also suggesting further empirical research involving key stakeholders. This will help guide the responsible design and successful implementation of wearable-based OHCA detection.

## METHODS

### Search Strategy and Selection Criteria

All data and supporting materials have been provided with the published article. This review was preregistered at the PROSPERO international register of systematic reviews (CRD42022370906). Because the literature specific to ethical challenges with wearable-based OHCA detection is nonexistent, a broad search string was constructed on wearable health technologies used for patient monitoring or location tracking in different health care settings, which could be applied to the case of wearable-based OHCA detection. The search string was constructed in collaboration with a librarian and consisted of a combination of three major concepts: ethics, health, and wearables. These were expanded with related terms, such as (1) moral, privacy, and bias; (2) health care, cardio, and medical; and (3) smartwatch, tracking, and mHealth. The entire search string can be found in Table S1. PubMed, Web of Science, and CINAHL were searched for academic literature published up to December 2023. Articles were included or excluded based on the criteria in Table 1.

**Table 1.**
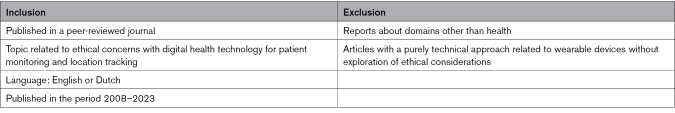
Inclusion and Exclusion Criteria of the Systematic Literature Search

Search results from the three databases were merged in EndNote 20, where duplicates were removed. A total of 7033 articles were screened by the first author on title and abstract in AS Review.^14^ After the initial selection, the remaining 169 papers were screened in full text by two authors (ME and MARB) using Covidence, a web-based collaboration software tool for literature reviews. Reference lists of the included articles were searched to find additional relevant literature. After the article selection, relevant ethical challenges in the literature were labeled based on the four principles' approach of Beauchamp and Childress.^15^ These principles (i.e., beneficence, nonmaleficence, autonomy, and justice) provide a comprehensive and widely used framework for analyzing and addressing ethical challenges in health care. Preferred Reporting Items for Systematic Review and Meta-Analyses guidelines were followed, but the quality assessment was considered unsuitable because of the conceptual nature of the ethics literature; all other Preferred Reporting Items for Systematic Review and Meta-Analyses criteria were used for reporting findings.

## RESULTS

### Included Studies

**Figure 2.**
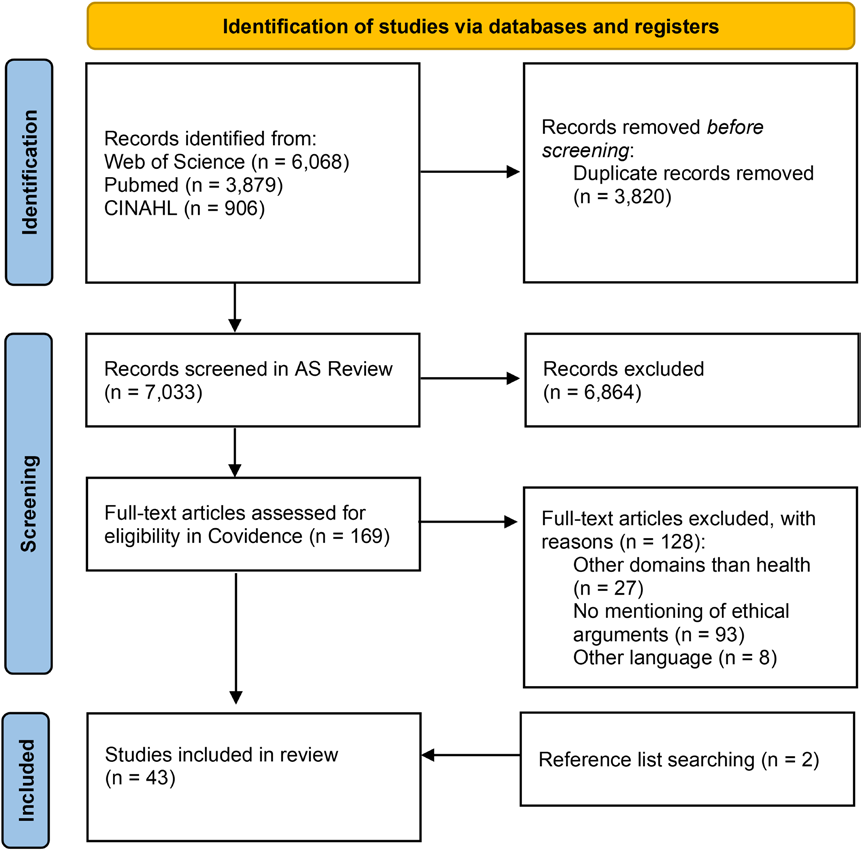
Flowchart of the study selection process.

A total of 43 articles were included in the final analysis (Figure 2). An overview of key study characteristics can be found in Table S2. A comprehensive overview of the identified challenges and potential solutions is given in Table 2.

**Table 2.**
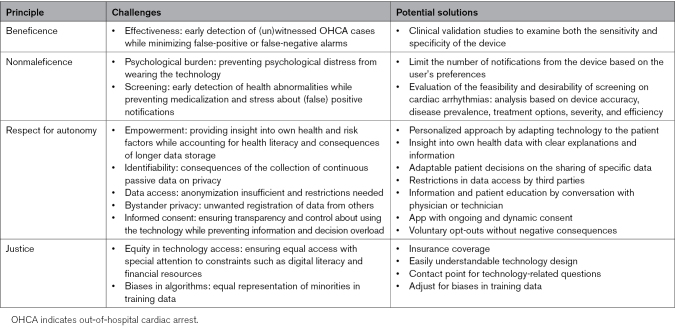
Overview of Ethical Challenges and Solutions for Wearable-Based OHCA Detection Derived From the Reviewed Literature

### Beneficence

#### Effectiveness

Benefits were often mentioned in terms of positive ethical values such as efficiency, health, and innovation. First, the immediate benefits of wearable health technology lie in their primary purpose. In the case of wearable-based OHCA detection, this would be to increase the chances of survival from OHCA, whereas location tracking systems in elderly care could help to retrieve patients with cognitive dysfunction (e.g., dementia) and wandering behavior.^16,17^ The effectiveness of wearable health technology is dependent on device accuracy and reliability.^18^ In wearable-based OHCA detection, this implies that clinical validation studies need to be conducted to examine both the sensitivity and specificity of the device. Both need to be high to lower the chances of false positives (e.g., an alert is sent to emergency responders without actual OHCA, which could overburden emergency dispatch centers) and false negatives (e.g., no alert during an actual OHCA, which raises questions of accountability). Next to the primary goal of detecting OHCA, the use of health technology data may also improve treatment outcomes of the patient by helping physicians in the early detection of health abnormalities, risk stratification, (early) health interventions, and may result in overall better health.^6,18,19^ The passive collection of health data by wearable technology is especially useful because it is considered more accurate than self-reported data.^20^ However, the extent to which physicians should monitor their patients’ health behavior data is debatable. The use of medical health technology could provide the clinician with an enormous amount of continuously collected data.^20^ However, the processing of all these data requires time, and there is a fear among health care professionals that an increase in technology use also results in an increase in work and changes to the workflow of the clinician.^19^ The feasibility of monitoring health technology data should be considered through careful assessment of the costs and benefits of how to effectively make use of these large amounts of data.

### Nonmaleficence

#### Psychological Burden

Wearable health technology is designed to be as comfortable and unobtrusive as possible.^21^ However, the presence of this technology for passive monitoring can also serve as a constant reminder of illness.^22^ This reminder is particularly present when a device interferes with the patient’s daily life, such as providing health notifications or messages with recommendations to contact a physician. These notifications could lead to stress or anxiety about the underlying cause, especially among more hypochondriacal individuals.^23,24^ For them, it might be desirable to receive fewer notifications because silent monitoring technologies can also be perceived as out of sight, out of mind.^16^ In addition, there is also a risk of becoming too dependent on the technology (e.g., physical or psychological burden when the technology is offline), making patients vulnerable to problems such as empty batteries, and software and network issues.^25,26^ There is a fine balance between too much and too little interference from the device, and it may vary from patient to patient whether screening, monitoring, or receiving feedback from wearable technology leads to better or worse psychological well-being.

#### Screening

The widespread usage of wearable technology might also open the door to large-scale screening for cardiac arrhythmias.^27,28^ The articles by Garikapati et al^29^ and Predel and Steger^30^ focused on the topic of screening, with an example of smartwatch-based screening for atrial fibrillation. Both articles mention that current device accuracy can result in extremely high numbers of false positives when screening for atrial fibrillation in the general population (i.e., 1 million false positives per 10 million people screened). Although the false-positive rates are lower when screening patients with earlier cardiac history (and higher a priori risk of atrial fibrillation), both studies conclude that more research and clinical validation studies need to be conducted to make wearable-based screening an ethically acceptable solution for early detection of atrial fibrillation. Aside from the arguments on accuracy, other ethical arguments have been raised against screening for arrhythmias. Screening can be problematic in the sense that a false-positive alarm may cause unnecessary stress among patients and take valuable time from health care professionals if they act upon these notifications.^30–32^ This is in accordance with Bayoumy et al.,^6^ who mention that inaccurate data as a result of technical challenges in accuracy and validity could be more harmful than no data. In the case of wearable-based screening for atrial fibrillation, it would put increased pressure on the health care system to assess all (false) positive notifications.

### Respect for Autonomy

#### Empowerment

Access to the data from wearable technology can increase the patient's self-knowledge about their illness or physical condition, potentially empowering them toward healthier behavior and a strengthened motivation to continue therapy.^20,33,34^ However, enacting and maintaining behavior change is difficult, and the added value of wearables in guiding behavior change is questioned.^6^ The extent to which the technology could aid patients to engage in long-term healthier behavior has yet to be researched. It has been suggested that technology should strengthen the patient-clinician relationship instead of replacing it and that mutual expectations about the usage of technology and collected data should be clear.^25^ Besides increasing insight into personal health behavior data, the use of wearable health technology might also empower the patient through improved psychological quality of life. Knowing that your medical condition is monitored by a device might potentially lead to a higher sense of security and might ease the worries that a patient may have about his or her condition, but the psychological effects of wearable-based OHCA detection have yet to be researched.

#### Identifiability

Due to the enormous amount of continuously and passively collected data when using wearable health technology, anonymization of user data in aggregated data sets is often considered insufficient.^23,35,36^ Depending on the amount and types of data being collected, data can often easily be reidentified. Fuller et al.^37^ argue that it is impossible to fully anonymize or deidentify sensor-based location and physical activity data due to the high velocity and volume of data being collected. As an example, the authors included a heat map of one week of traveling by the lead author, which gives a detailed visualization of his whereabouts (e.g., residence and workplace). Although this extent of personal disclosure could be considered undesirable in the general population, the use of real-time location systems might be justified in situations where further harm could be prevented, such as a solution to wandering behavior for people with dementia.^16,17^ Other examples, such as measuring relative spatial proximity in COVID-19 contact tracing apps, are less privacy-invasive than live location tracking but still pose a risk of reidentification of individuals or groups.^38–42^ In the case of wearable-based OHCA detection, it is important to consider the effects of continuous location monitoring against the consequences of only sending a GPS location if the algorithm detects a cardiac arrest. Alerting emergency responders after detecting OHCA only requires a location of that moment in time, while continuous location monitoring could be useful for analyzing individual movement patterns that might predict OHCA. The processing of these data needs to be adequate, relevant, and limited to what is necessary in relation to the purposes, which is important to consider when balancing out the benefits of the technology against the violation of privacy.^21^

#### Data Access

Due to the higher identifiability related to continuous data generation through smartphone apps and wearable technology, in the selected articles, it was questioned which parties have access to the data and what they will do with the data. Whereas health care providers are expected to put the interest of the patient first, technology development companies act more from a commercial interest.^43,44^ This raises concerns among users about behavioral data being sold and used to classify people, which could lead to potential harms such as discriminatory profiling and manipulative marketing.^22,45^ On the other hand, in articles on smartphone apps for preventing the spread of communicable diseases, which are frequently implemented by governmental institutions, ethical considerations focus on the risks of mass government surveillance and balancing the right to be left alone against the benefits of public health.^42,46,47^ This highlights that both the goal and the nature of the manufacturer of the technology result in contextually different challenges and concerns. Still, there are also more general data protection principles that apply to all forms of wearable health technology. These include transparency, confidentiality, consent, and limitation of purpose, data, and storage, which are key elements of the General Data Protection Regulation.

#### Bystander Privacy

When data are collected continuously, both users and their surroundings might experience situations where data are gathered about events that they do not want to share with others. Perez and Zeadally^48^ mention the issue of bystander privacy, where other people are affected by the use of a wearable device in their surroundings.^48^ Common examples are the use of cameras (e.g., smart glasses) and microphones (e.g., voice assistants) that might capture the behavior of other people without their consent. Mentioned solutions to these privacy issues involve mechanisms for allowing or objecting to data collection, notification systems, and blurring of people in surroundings. In the case of wearable-based OHCA detection, bystander privacy is especially important. Examples of unwanted registration of behavioral data could be patterns in sleep and daily activity that would provide physicians with a lot of intimate knowledge about patients’ personal lives and the lives of their partners who might not have consented to share this information. Also, the decision to share the home address in advance in a citizen responder smartphone app, which could facilitate the localization of the OHCA victim, has consequences for the privacy of people living at the same address. The question of whether the partner therefore has a say in these decisions was not covered in the literature and should be further studied through normative ethical analysis and empirical study among next-of-kin.

#### Informed Consent

Various articles criticized the process of giving informed consent for data collection by existing wearable health technologies. Patients are often informed through long documents that spell out the terms under which the technology can be used and what may be done with the acquired data.^25^ However, because of their length, these documents are rarely read.^22,49^ Van Hoof et al.^16^ raise the question of whether consent really represents a full state of being informed in these cases. Next to that, patients may feel that they have no choice but to accept, given that these consent forms are often not negotiable and not agreeing means not receiving the device.^25^

It will be especially difficult to opt out when these devices are becoming further integrated into the way people engage with care providers and other institutions.^45^ This asks for a change in the way patients should give informed consent when dealing with passive health monitoring.^43,50^ Asking permission for each instance of data collection is unfeasible and difficult to convey in an informed consent form.^37,45^ Measures of dynamic consent, in which data recording can be paused or data can be deleted by the user after recording, could be a feasible solution to deal with the specificity and volume of data being collected.^25,37^ These systems could, for example, be built into smartphone apps that manage user preferences, where permission to share data could easily be switched on and off.^51^

### Justice

#### Equity in Technology Access

An often-mentioned ethical challenge is the current unequal access to digital technology in general, the so-called digital divide. Patients vary greatly in their access to digital technology, the necessary technical skills, and their ability to safely use the technology.^19,22,52,53^ These variations may be due to factors such as income, age, education, internet access, or geographic restrictions. As a result, the use of wearable health technology is currently skewed toward those who may need the least medical help, being the young, the fit, and the highly educated.^54^ At the same time, the elderly or those with low incomes could be restrained in their technological access.^43,45,55,56^ Paradoxically, the groups with the highest health care needs often have the least access to digital health technology, which could lead to exacerbating health inequities when unwillingly missing out on health benefits.^55^ These inequities ask for strategies to mitigate perceived barriers in addressing this digital divide. Actions could involve easy-to-implement design choices to overcome barriers related to size, display visibility, battery life, or lack of a waterproof design.^57^ However, design choices on their own might not be enough to close the digital divide, and broader societal strategies to promote digital equity and inclusion are needed. Especially concerning financial constraints, considering affordability as a principal component of making wearable technology accessible is needed to achieve successful integration into society.

#### Biases in Algorithms

Another justice concern is the underrepresentation of minority groups in the training data of health technology applications. If not accounted for, inadequate representation could result in algorithms under or overperforming for specific groups. A clear example is the photoplethysmography signal in wearable technology that measures pulsatile blood flow and may respond differently depending on skin tone. As a consequence, algorithms for wearable-based monitoring, which were developed based on data obtained from people with a light skin tone, perform less well among people with a darker skin tone.^30^ In the case of wearable-based OHCA detection, this would result in more false positives (i.e., alarm in the absence of a cardiac arrest) or false negatives (i.e., no alarm during an actual cardiac arrest) among people with a darker skin tone, which is highly discriminatory and undesirable. These flaws point toward the need for more diverse training data, with regard to age, sex, and skin tone. In addition, a partial solution could be to adjust the power of the LED lights in wearable technology to ensure the same light reflection among various skin tones to strive for the same quality of care for everyone.

## DISCUSSION

This review identified ethical challenges with wearable-based cardiac monitoring and location tracking relevant to the wearable-based detection of OHCA. Major themes were expected costs and benefits (related to device accuracy and reliability, the well-being of the patient, and the integration of wearable technology in the clinical workflow of the physician and other health care professionals), as well as autonomy and justice concerns (related to the personal identifiability from the data and consent procedures with regard to sharing data, making the technology accessible for everyone with an increased risk of OHCA, and minimizing bias in the training data sets). Compared with an earlier review by Maher et al.^12^ on passive data collection in health care, we found more emphasis on broader ethical challenges (i.e., beyond privacy and data protection issues), such as user experiences, screening, consent procedures, and technological literacy. The literature on smartphone-based medical devices raises similarly broad ethical challenges, with a critical reflection on promises of efficiency, empowerment, accessibility, and social justice.^58,59^ Of note is that a fundamental difference between wearable applications and smartphone-based medical devices is the interaction between the user and the device, with subsequent consequences for psychological well-being and privacy. Wearing a device on your arm could create a stronger reminder of illness than having a smartphone in your pocket. Still, there could also be less interference because data are collected more passively and continuously, which might lead to a state where a person forgets being monitored, which then raises questions of privacy. Empirical research into user experiences will teach us more about these specific mechanisms, which might differ between various types of wearable technology, potentially as a result of the interaction between the technology and the user.

Despite our extensive literature search, some ethical challenges that we think are important for the successful implementation of wearable-based OHCA detection were mentioned to a limited extent in the literature, for instance, the effects of false positives (e.g., an alert is sent to emergency responders without actual OHCA), false negatives (e.g., no alert during an actual OHCA), and difficulties in gaining access to an OHCA victim who is alone but located in a locked room (e.g., at home). These different challenges highlight the need for a case-specific identification of ethical challenges and the potential burden of the technology on the health care system when implemented. Therefore, in the next section, we will make recommendations specific to the ethical challenges with wearable-based OHCA detection based on the more general solutions mentioned in the reviewed literature.

### Design Recommendations for Wearable-Based OHCA Detection

Solutions to the ethical challenges need to be incorporated into the design of the wearable technology as far as possible and require further research, as well as reflection on ethically relevant aspects in the implementation phase (Table 3). Combining the results from the reviewed literature, we suggest four key design recommendations that developers of wearable-based OHCA detection should focus on: device accuracy and reliability, dynamic consent, purpose limitation, and personalization. First, device accuracy and reliability are important for creating a device that is trustworthy for both the patient and the people acting upon the alarms, which requires strong clinical validation studies before implementation into society and a thorough minimization of false positives to avoid unnecessary ambulance dispatching. Second, dynamic consent involves an online consent platform providing patients with a free and informed choice about using the technology with the possibility to continuously decide which data are shared when and with whom. Third, purpose limitation requires the device to only collect and store data necessary to detect OHCA to limit privacy invasions. With regard to additional functionalities, screening for atrial fibrillation within OHCA detection devices should be postponed until those algorithms reach lower false positive rates and, even then, may not be included at all after further ethical-legal reflection.^29,30^ This reflection entails that existing guidelines on the feasibility of screening need to be considered, such as the criteria by Wilson and Jungner, which set criteria such as prediction accuracy, disease prevalence and severity, and the availability of treatment options.^60^ Fourth, personalization covers both the possibility to adapt the technology to the wearer (e.g., providing insight into health data or limiting the number of notifications), as well as promoting the accessibility of the technology for everyone at risk of OHCA. Important constraints in the digital divide are insufficient digital literacy and insufficient financial resources.^61^ Attempts should be made to lower these thresholds specifically for disadvantaged potential users: easy-to-understand smartphone apps or assigning a contact person for technology-related questions could help people with lower digital literacy, whereas complete insurance coverage could help people with lower financial resources.^62^

**Table 3.**
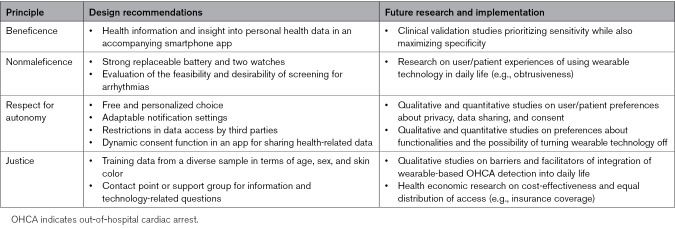
Design Recommendations and Future Research Specific to Wearable-Based OHCA Detection

Some disclaimers are in place about our design recommendations. The propositions to ethical challenges need to be considered in conjunction with each other, especially because these propositions may conflict with each other, which requires careful deliberation. For example, adaptable privacy settings would increase autonomy in the sharing of data but also make the technology more complicated and, therefore, less accessible for people with lower digital literacy. The adverse consequences of technological solutionism need to be minimized to avoid that every technology-related problem will be solved with a technological fix.^63^ While complementing wearable technology with a smartphone app could be a valuable solution to tackle some of the ethical challenges, designers also need to consider that there are still people without smartphones, especially among the elderly.^64^ In this case, less technology might be the answer by facilitating the use of wearable technology with predetermined privacy settings and no involvement of smartphone apps. Another area for future research is the scalability of the device implementation and the related economic cost analysis. The different types of stand-alone wearable devices, which are either specifically designed or consist of an algorithm integrated into existing wearables, as well as the choice of a smartphone app, will all have different implications for the costs of the technology, both from an individual perspective (e.g., affordability) and a societal perspective (e.g., the burden of false alarms), in which an optimal balance should be sought.

### Regulatory Requirements and Further Research on Psychosocial Impact

Our review started with the claim that the ethics part of ethical, legal, and social issues should form the basis for further legal analyses and empirical studies investigating psychosocial impacts. This section covers relevant legislation and future research on the psychosocial impact of using the technology. Current efforts on the development of wearable-based OHCA detection are mostly based in Europe, where the first step to market access is obtaining a CE marking.^65^ Wearable health technology with an intended medical purpose is regulated by the European Medical Device Regulation.^66,67^ Because the use of wearables involves the collection and processing of large amounts of sensitive personal health data, data protection legislation, such as the General Data Protection Regulation and national health law that includes provisions on health data, comes into play.^68^ In addition, medical devices are classified as high-risk systems in the AI Act, which implies adherence to stricter requirements.

There are two questions that should be answered before it becomes clear what the possible impact of the legislation is: first, whether the scope of the European Medical Device Regulation and the AI Act extends to the wearable technology in question; second, what the purpose of data collection is. If the goal of the collection would only be to detect and alert emergency responders during OHCA, then storing the data beyond the OHCA event would not be justified without the individual’s authorization. However, if the purpose is broadened to telemonitoring or screening, long-term storage of data is required. Specific guidelines or professional codes are necessary to support the further implementation and data protection of wearable technology in these specific contexts, in particular when devices are developed together with private parties that often have commercial interests.

Further research is necessary for the development of such codes and the integration of wearable-based OHCA detection into society. In the case of the BECA project in the Netherlands, this will involve stakeholders such as cardiology patients with an increased risk of OHCA, their significant others, emergency responders, and health care professionals.^9^ Improvements in device accuracy are also important as these could reduce the number of false-positive and false-negative notifications, which also impacts the psychological and ethical aspects of using the device. The reviewed articles stated that screening for arrhythmia within the context of OHCA detection devices is not feasible at the moment, but technological developments in device accuracy could make such screening possible in the future. However, even if technologically feasible, this option should also be studied from a normative point of view, focusing on ethical and legal desirability.

## CONCLUSIONS

This review identified ethical challenges with wearable-based OHCA detection, which need to be addressed before and during implementation into society and clinical practice. The initial major challenge with OHCA detection and device accuracy is to prioritize sensitivity while simultaneously maximizing specificity to prevent overburdening the emergency medical service infrastructure and stress among users as a result of false positive alarms. Clinical validation studies are, therefore, needed to assess and improve device accuracy. Simultaneously, quantitative and qualitative studies on user experiences should study the effects of the technology on the well-being of the individual patient. We find that broadening the purpose of the device, for instance, screening for atrial fibrillation, should be postponed until normative and technical questions are sorted out. In this article, we made specific design recommendations, and we argued that efforts should be made to make the technology inclusive and accessible for those who could benefit from it. Further empirical research and an open debate between various stakeholders and experts on the final design and implementation of wearable-based OHCA detection will ensure optimal quality and integration of this technology into society and clinical practice.

## ARTICLE INFORMATION

### Acknowledgments

The authors express their appreciation to D. Rutten (Head of Research Support, Library, Tilburg University) for providing guidance in constructing the literature search string.

### Sources of Funding

Eversdijk, Habibović, Willems, Kop, Dekker, Tan, Vullings, and Bak are involved in the Beating Cardiac Arrest (BECA) project. This project was supported by the PPP Allowance made available by Top Sector Life Sciences and Health to the Dutch Heart Foundation to stimulate public-private partnerships (grant 01-003-2021-B005; BECA project).

### Disclosures

None.

### Supplemental Material

Tables S1 and S2

## Supplementary Material
