# Supplementary material for: Ethics of Wearable-Based Out-of-Hospital Cardiac Arrest Detection
Source: Circ Arrhythm Electrophysiol. 2024 Aug 22;17(9):e012913. doi: 10.1161/CIRCEP.124.012913 (PMC11410148; doi:10.1161/CIRCEP.124.012913)
Supplement: Supplementary file 1 [file hae-17-e012913-s001.pdf]

SUPPLEMENTAL MATERIAL

Table S1. Overview of search strings and results

Source	Search string	Results
**Web of science**	((AB=(Ethic* OR moral* OR privacy OR informed consent OR confidentiality OR bias OR discrimination)) AND AB=(health OR care OR healthcare OR cardi* OR heart OR arrhythm* OR medical OR incidental findings)) AND AB=(wearable* OR smartwatch OR tracking OR tracing OR biometric data OR mhealth)<br><br>((TI=(Ethic * OR moral* OR privacy OR informed consent OR confidentiality OR bias OR discrimination)) AND TI=(health OR care OR healthcare OR cardi* OR heart OR arrhythm* OR medical OR incidental findings)) AND TI=(wearable* OR smartwatch OR tracking OR tracing OR biometric data OR mhealth)	6,068 articles
**Pubmed**	(Ethic* [tiab] OR moral* [tiab] OR privacy [tiab] OR informed consent [tiab] OR confidentiality [tiab] OR bias [tiab] OR discrimination [tiab]) AND (health [tiab] OR care [tiab] OR healthcare [tiab] OR cardi* [tiab] OR heart [tiab] OR arrhythm* [tiab] OR medical [tiab] OR incidental findings [tiab]) AND (wearable* [tiab] OR smartwatch [tiab] OR tracking [tiab] OR tracing [tiab] OR biometric data [tiab] OR mhealth [tiab])	3,879 articles
**CINAHL**	AB (Ethic * OR moral* OR privacy OR informed consent OR confidentiality OR bias OR discrimination) AND AB (health OR care OR healthcare OR cardi* OR heart OR arrhythm* OR medical OR incidental findings) AND AB (wearable* OR smartwatch OR tracking OR tracing OR biometric data OR mhealth)<br><br>TI (Ethic * OR moral* OR privacy OR informed consent OR confidentiality OR bias OR discrimination) AND TI (health OR care OR healthcare OR cardi* OR heart OR arrhythm* OR medical OR incidental findings) AND TI (wearable* OR smartwatch OR tracking OR tracing OR biometric data OR mhealth)	906 articles

Table S2. Article overview

Nr.	First author	Title	Year	Country	Focus	Ethical principle
1.	Akinsanmi, T.	Evaluating the trade-off between privacy, public health safety, and digital security in a pandemic.	2021	Nigeria, South Africa, Canada	Contact tracing apps against the spread of COVID-19	Autonomy
2.	Anaya, L. H. S.	Ethical Implications of User Perceptions of Wearable Devices.	2018	Australia	Wearable health technology in general	Autonomy
3.	Bauer, M.	Ethical perspectives on recommending digital technology for patients with mental illness.	2017	Germany	Smartphone apps for monitoring mental illnesses	Beneficence; Autonomy; Justice
4.	Bayoumy, K.	Smart wearable devices in cardiovascular care: where we are and how to move forward.	2021	United States, Qatar	Smart sensors in cardiology	Beneficence; Non-maleficence
5.	Brandes, A.	Consumer-Led Screening for Atrial Fibrillation: Frontier Review of the AF-SCREEN Internation Collaboration	2022	United States	Smart sensors in cardiology	Non-maleficence
6.	Canali, S.	Wearable Technologies and Stress: Toward an Ethically Grounded Approach	2023	Italy	Wearable health technology in general	Beneficence; Non-maleficence; Autonomy; Justice
7.	Cvrkel, T.	The ethics of mHealth: Moving forward.	2018	United States	Wearable health technology in general in medical care	Beneficence; Non-maleficence
8.	Chung, J.	Community-dwelling older adults' acceptance of smartwatches for health and location tracking	2022	United States	Wearable health technology in general	Beneficence; Autonomy
9.	Esmonde, K.	A social and ethical framework for providing health information obtained from combining genetics and fitness tracking data	2023	United States	Wearable health technology in general	Autonomy; Justice
10.	Ford, E.	Ethical issues when using digital biomarkers and artificial intelligence for the early detection of dementia	2023	United Kingdom	Smart sensors in geriatrics	Non-maleficence
11.	Fuller, D.	Ethical implications of location and accelerometer measurement in health research studies with mobile sensing devices.	2017	Canada, United Kingdom	Wearable health technology in general	Autonomy
12.	Garikapati, K.	The Role of Contemporary Wearable and Handheld Devices in the Diagnosis and Management of Cardiac Arrhythmias.	2022	Australia	Smart sensors in cardiology	Non-maleficence
13.	Gasser, U.	Digital tools against COVID-19: taxonomy, ethical challenges, and navigation aid.	2020	United States, Switzerland	Contact tracing apps against the spread of COVID-19	Autonomy
14.	Kilgallon, J. L.	Passive Data Use for Ethical Digital Public Health Surveillance in a Postpandemic World.	2022	United States, Netherlands	Wearable health technology in general in medical care	Justice
15.	Klugman, C. M.	The Ethics of Smart Pills and Self-Acting Devices: Autonomy, Truth-Telling, and Trust at the Dawn of Digital Medicine.	2018	United States	Wearable health technology in general	Beneficence; Non-maleficence; Autonomy
16.	Landau, R.	Ethical aspects of using GPS for tracking people with dementia: recommendations for practice	2012	Israel	Smart sensors in geriatrics	Beneficence; Autonomy
17.	Leclercq, C.	Wearables, telemedicine, and artificial intelligence in arrhythmias and heart failure:	2022	France, Germany, United States,	Smart sensors in cardiology	Beneficence; Non-maleficence; Justice

		Proceedings of the European Society of Cardiology Cardiovascular Round Table.		Switzerland, United Kingdom, Sweden, Austria		
18.	Lee, H.	Toward Dynamic Consent for Privacy-Aware Pervasive Health and Well-being: A Scoping Review and Research Directions	2022	South Korea	Wearable health technology in general	Autonomy
19.	Mann, M.	Between surveillance and technological solutionism: A critique of privacy-preserving apps for COVID-19 contact-tracing.	2022	Australia	Contact tracing apps against the spread of COVID-19	Autonomy
20.	Martinez-Martin, N., Luo, Z.	Ethical issues in using ambient intelligence in health-care settings.	2021	United States	Wearable health technology in general in medical care	Autonomy; Justice
21.	Martinez-Martin, N., Greely, H. T.	Ethical Development of Digital Phenotyping Tools for Mental Health Applications: Delphi Study.	2021	United States	Wearable health technology in general in medical care	Autonomy
22.	Mbunge, E.	Ethics for integrating emerging technologies to contain COVID-19 in Zimbabwe.	2021	Swaziland, South Africa	Contact tracing apps against the spread of COVID-19	Autonomy
23.	Mittelstadt, B.	Ethics of the health-related internet of things: a narrative review	2017	United Kingdom	Wearable health technology in general in medical care	Beneficence; Autonomy
24.	Montgomery, K.	Health Wearables: Ensuring Fairness, Preventing Discrimination, and Promoting Equity in an Emerging Internet-of-Things Environment.	2018	United States	Wearable health technology in general	Autonomy; Justice
25.	Müller, R.	Ethical, legal, and social aspects of symptom checker applications: a scoping review	2022	Germany	Wearable health technology in general	Beneficence; Non-maleficence; Autonomy; Justice
26.	Müller, R.	Ethics of sleep tracking; techno-ethical particularities of consumer-led sleep-tracking with a focus on medicalization, vulnerability, and relationality	2023	Germany	Wearable health technology in general	Beneficence; Non-maleficence; Autonomy
27.	Pagliari, C.	The ethics and value of contact tracing apps: International insights and implications for Scotland's COVID-19 response.	2020	United Kingdom	Contact tracing apps against the spread of COVID-19	Autonomy
28.	Patel, S.	Apps and wearables in the monitoring of mental health disorders.	2018	United Kingdom	Smartphone apps for monitoring mental illnesses	Justice
29.	Perez, A. J.	Privacy Issues and Solutions for Consumer Wearables.	2018	United States	Wearable health technology in general	Autonomy
30.	Predel, C.	Ethical Challenges With Smartwatch-Based Screening for Atrial Fibrillation: Putting Users at Risk for Marketing Purposes?	2020	Germany	Smart sensors in cardiology	Non-maleficence; Justice
31.	Predel, C.	Conflicting Aims and Values in the Application of Smart Sensors in Geriatric Rehabilitation: Ethical Analysis.	2022	Germany	Smart sensors in geriatrics	Beneficence
32.	Ranisch, R.	Digital contact tracing and exposure notification: ethical guidance for trustworthy pandemic management.	2021	Germany, Singapore, Sweden	Contact tracing apps against the spread of COVID-19	Autonomy

33.	Rozier, M.	Personal Location as Health-Related Data: Public Knowledge, Public Concern, and Personal Action	2023	United States	Wearable health technology in general	Autonomy
34.	Schmietow, B.	Mobile health ethics and the expanding role of autonomy.	2019	Germany	Wearable health technology in general	Justice
35.	Singhal, A.	Digital Health: Implications for Heart Failure Management.	2021	United Kingdom	Smart sensors in cardiology	Justice
36.	Spears, J. L.	Privacy risk in contact tracing systems.	2021	United States, Sweden	Contact tracing apps against the spread of COVID-19	Autonomy
37.	Sui, A.	Ethical considerations fort he use of consumer wearables in health research	2023	Canada	Wearable health technology in general	Autonomy
38.	Tchapmi, D. P.	The use of digital health in heart rhythm care	2023	United States	Smart sensors in cardiology	Non-maleficence; Equity
39.	Ullah, M.	Smart Technologies used as Smart Tools in the Management of Cardiovascular Disease and their Future Perspective	2023	Pakistan, South Korea	Smart sensors in cardiology	Beneficence; Non-maleficence; Autonomy
40.	van Hoof, J.	Real-Time Location Systems for Asset Management in Nursing Homes: An Explorative Study of Ethical Aspects.	2018	The Netherlands, Poland	Smart sensors in geriatrics	Beneficence; Autonomy
41.	Wangmo, T.	Ethical concerns with the use of intelligent assistive technology: findings from a qualitative study with professional stakeholders.	2019	Switzerland	Smart sensors in geriatrics	Justice
42.	Wieczorek, M.	The ethics of self-tracking. A comprehensive review of the literature.	2022	Ireland, The Netherlands	Wearable health technology in general	Beneficence; Autonomy
43.	Xue, Y. K.	A review on intelligent wearables: Uses and risks.	2019	United States	Wearable health technology in general	Beneficence
